# Identification of a Potential PPAR-Related Multigene Signature Predicting Prognosis of Patients with Hepatocellular Carcinoma

**DOI:** 10.1155/2021/6642939

**Published:** 2021-03-12

**Authors:** Wenfang Xu, Zhen Chen, Gang Liu, Yuping Dai, Xuanfu Xu, Duan Ma, Lei Liu

**Affiliations:** ^1^Department of Biochemistry and Molecular Biology, School of Basic Medical Sciences and Institutes of Biomedical Sciences, Fudan University, Shanghai, China; ^2^Department of Pathology, Shidong Hospital, Yangpu District, Shanghai, China; ^3^Department of Gastroenterology, Shidong Hospital, Yangpu District, Shanghai, China

## Abstract

Peroxisome proliferator-activated receptors (PPARs) and part of their target genes have been reported to be related to the progression of hepatocellular carcinoma (HCC). The prognosis of HCC is not optimistic, and more accurate prognostic markers are needed. This study focused on discovering potential prognostic markers from the PPAR-related gene set. The mRNA data and clinical information of HCC were collected from TCGA and GEO platforms. Univariate Cox and lasso Cox regression analyses were used to screen prognostic genes of HCC. Three genes (*MMP1*, *HMGCS2*, and *SLC27A5*) involved in the PPAR signaling pathway were selected as the prognostic signature of HCC. A formula was established based on the expression values and multivariate Cox regression coefficients of selected genes, that was, risk score = 0.1488∗expression value of *MMP*1 + (−0.0393)∗expression value of *HMGCS*2 + (−0.0479)∗expression value of *SLC*27*A*5. The prognostic ability of the three-gene signature was assessed in the TCGA HCC dataset and verified in three GEO sets (GSE14520, GSE36376, and GSE76427). The results showed that the risk score based on our signature was a risk factor with a HR (hazard ratio) of 2.72 (95%CI (Confidence Interval) = 1.87 ~ 3.95, *p* < 0.001) for HCC survival. The signature could significantly (*p* < 0.0001) distinguish high-risk and low-risk patients with poor prognosis for HCC. In addition, we further explored the independence and applicability of the signature with other clinical indicators through multivariate Cox analysis (*p* < 0.001) and nomogram analysis (C‐index = 0.709). The above results indicate that the combination of *MMP1*, *HMGCS*2, and *SLC*27*A*5 selected from the PPAR signaling pathway could effectively, independently, and applicatively predict the prognosis of HCC. Our research provided new insights to the prognosis of HCC.

## 1. Introduction

Liver cancer is a common malignancy and its mortality rate ranks fourth among cancer-related deaths [1]. About 80% of patients with primary liver cancer belong to the hepatocellular carcinoma (HCC) category. HCC is a rapidly developing disease with poor prognosis. Currently, less than 18% of HCC patients have an overall survival (OS) time of more than 5 years [2]. In addition, due to the heterogeneity and the lack of effective prognostic markers for HCC, it is difficult to accurately predict the prognosis of patients with HCC [3, 4]. It is urgent to study the prognostic markers of HCC to ensure that patients could receive more appropriate and effective treatment.

For many cancer types, the identification of specific molecular markers can solve the problem of prognosis differentiation caused by tumor heterogeneity and provide patients with more suitable and effective treatment. For example, the *KRAS* gene mutation shows a high prediction accuracy for the prognosis of patients with metastatic colorectal cancer [5], and accumulated studies have established that the methylation level of the promoter of *MGMT* can be used to predict the efficacy of temozolomide in patients with glioma [6]. However, there are currently no available molecular markers for HCC in clinical applications.

Peroxisome proliferator-activated receptors (PPARs) are nuclear receptors as transcription factors that regulate physiological activities such as invasion, immune tolerance, metabolism, and inflammation [7, 8]. Numerous studies have revealed that tumorigenesis and cancer progression are usually accompanied by abnormal regulation of the PPAR signaling pathway [9–12]. In addition, in recent studies on HCC prognostic markers, it has been repeatedly reported that the PPAR signaling pathway is dysregulated in high-risk HCC patients with poor prognosis [13–15]. Although the PPAR signaling pathway has been reported as one of the prognostic characteristic pathways of HCC, no one has screened the prognostic markers for HCC from the genes involved in this pathway.

In the context of the above research, this study was dedicated to select a prognostic multigene biomarker in HCC from PPAR-related genes. Based on 365 HCC samples included in TCGA, we analyzed the correlation between the mRNA levels of 69 PPAR-related genes and the overall survival of patients. A combination of three genes (*MMP1*, *HMGCS2*, and *SLC27A5*) was selected as a prognostic marker. Next, the performance of the prognostic marker was evaluated and validated in three validation sets from the GEO database. At the same time, the effects of this marker and other clinical indicators on the OS of HCC were analyzed and compared. Finally, a nomogram was developed to provide the possibility of clinical application of the prognostic multigene biomarker.

## 2. Materials and Methods

### 2.1. Sample Acquisition and Data Preprocessing

The 365 primary HCC samples with survival information in the TCGA cohort were selected as the training set. The level 3 values of mRNA and corresponding clinical data of HCC were collected from Xena, University of California, Santa Cruz (UCSC) database. The values of gene expression were the counts obtained by the RSEM algorithm. Used UCSC Xena HUGO probeMap to map genes to reference genomes. For details of the processing method, please refer to the website of the TCGA Genome Characterization Center of the University of North Carolina. In addition, removed low-expressed genes that were not expressed in more than 75% of patients and whose average values of expression were less than 1.

The validation sets were three HCC datasets in the GEO database: GSE14520 (*n* = 221), GSE36376 (*n* = 223), and GSE76427 (*n* = 115) (*n* represents the number of samples). We chose the normalized mRNA data. For details, please refer to the “_series_matrix.txt” files of the three datasets in GEO. Gene annotation was completed according to the annotation files provided by the microarray sequencing platforms (that is, GSE14520 corresponds to GPL3921, and GSE36376 and GSE76427 both correspond to GPL10558). When a gene matched multiple probes, the average expression value of multiple probes was selected as the expression value of the gene.

In addition, 69 PPAR signaling pathway-related genes were obtained from the Kyoto Encyclopedia of Genes and Genomes (KEGG) database (KEGG pathway ko03320).

### 2.2. Screening and Evaluating Prognostic Genes

To find an efficient prognostic gene combination for HCC from 69 genes related to the PPAR signaling pathway, firstly, based on the expression values of these 69 genes, we used the univariate Cox regression algorithm to analyze the OS of HCC patients. The genes with *p* < 0.05 were considered as genes related to the OS of HCC. Subsequently, based on the candidate genes selected in the previous step, using the lasso Cox regression algorithm, and applying tenfold cross-validation to select the best penalty coefficient, the best combination of HCC prognostic genes could be obtained [16].

To evaluate the effectiveness of the prognostic gene combination we selected, we used Equation (1) to establish a prognostic model:

(1) $$\begin{array}{c} \text{Risk score}=\overset{n}{\underset{i}{\sum }}x_{i}*\beta_{i}, \end{array}$$

where *x*_*i*_ indicates the expression value of gene *i*; meanwhile *β*_*i*_ means the coefficient of gene *i* generated from the multivariate Cox regression analysis. The risk score of each HCC sample was calculated according to Equation (1), and the samples were divided into high- and low-risk groups according to the median value of the risk score. To assess the survival difference between the two groups to show the efficiency of our prognostic genes, a log-rank test analysis was performed. In addition, we evaluated the specificity and sensitivity of the multigene marker in predicting the 1-, 3-, and 5-year survival rates of HCC and compared them with other clinical indicators such as age, gender, AFP, and TNM staging, and the method used was a time-dependent receiver operating characteristic (ROC) curve. The predictive effect of the multigene marker eventually was verified in the GSE14520, GSE36376, and GSE76427 datasets.

### 2.3. Detecting the Independence of the Multigene Signature

To find out whether this multigene marker could be independent of other clinically commonly used prognostic indicators of HCC, we applied univariate and multivariate Cox regression analysis methods to analyze the survival of HCC patients. As for the clinical factors that might affect the prognosis of HCC, we selected six indicators: age, gender, AFP, TNM staging, histological grade, and vascular tumor invasion. Risk score and age were treated as continuous variables, while the remaining variables were categorical variables. Clinical indicators associated with survival were initially identified; then, the association between risk scores and other survival-associated clinical indicators was assessed with a log-rank test. A nomogram was constructed using those variables that were identified as independent predictors, which the predictions of 1-, 3-, and 5-year survival rates were corrected by correcting for the consistency between true and predicted values.

### 2.4. Statistical Methods

The R software (version 3.6.1) was used for all analyses in the present study. Microarray data were analyzed with the “GEOquery” package, while the “edgeR” package was employed for differential gene screening. The “survival::coxph” function was used to conduct univariate and multivariate Cox analyses, while lasso Cox regression analyses were performed with the web-based tool (ESurv) [17]. The “survdiff” function from the “survival” package was utilized for log-rank testing, and time-dependent ROC analyses were similarly conducted with the “timeROC” package. Heatmaps were prepared with the “ggplot::heatmap” function, while the “forestplot” package was used to generate forest plots, and the nomogram was established and implemented with the “rms” package.

## 3. Results

### 3.1. PPAR-Related Prognostic Genes for HCC

**Figure 1:**
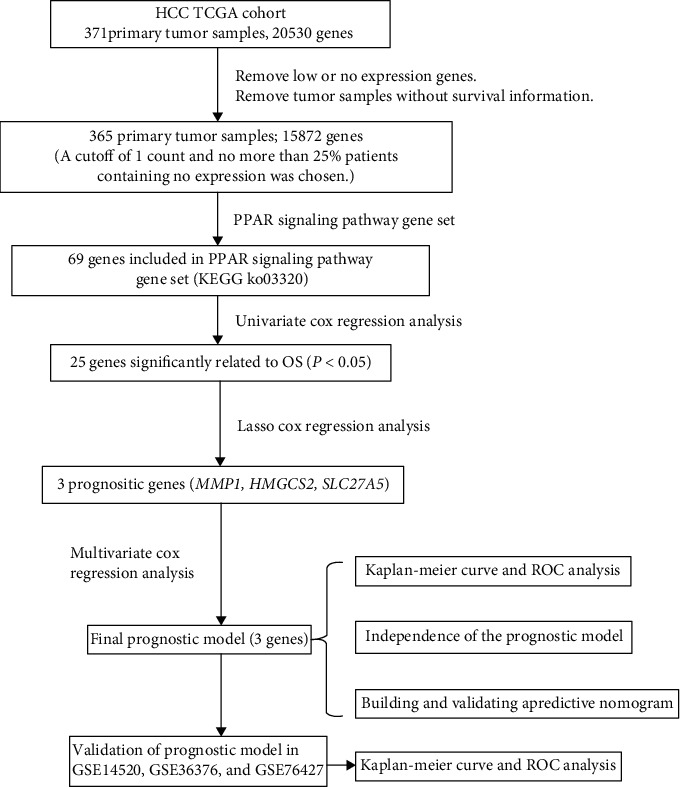
The flow chart about the study of PPAR-related gene signature in predicting survival of HCC.

**Figure 2:**
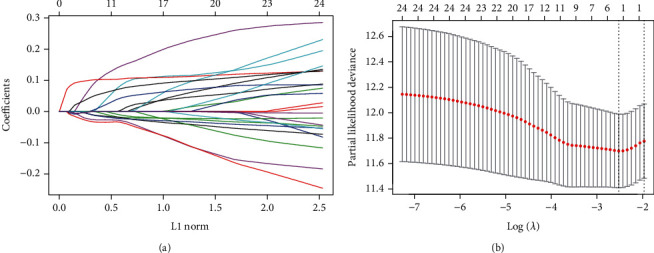
Prognostic gene screening was conducted through lasso Cox regression analyses. Positive and negative regression coefficients, respectively, correspond to positive and negative correlations between numbers (a). The best parameter (*λ*) in the lasso analysis was then selected (b).

Our study was carried out through the procedure which is shown in Figure 1. To determine genes related to the OS of HCC from the PPAR signaling pathway, we analyzed the transcriptome data of 365 primary HCC samples in TCGA and used univariate cox regression analysis. Twenty-five PPAR-related genes were identified as being related to the OS of HCC (*p* < 0.05). Finally, three prognostic genes (including *MMP1*, *HMGCS2*, and *SLC27A5*) were obtained by lasso Cox regression analysis from candidate prognostic genes (Figure 2).

### 3.2. Prognostic Model Establishment and Evaluation

**Figure 3:**
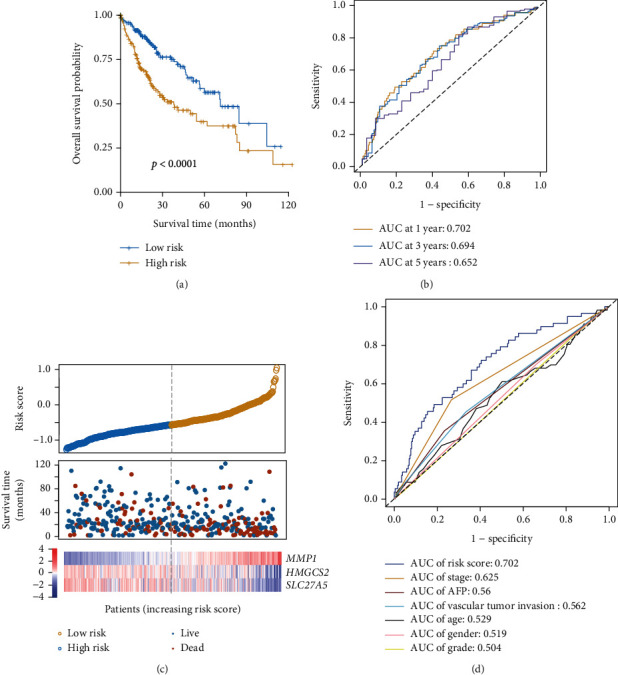
PPAR-associated prognostic genes were significantly associated with HCC patient overall survival. A Kaplan-Meier analysis of low- and high-risk patients in the TCGA HCC patient cohort, revealing a poorer prognosis for those with high-risk scores (a). A ROC analysis of risk scores was performed to assess their sensitivity and specificity (b). The relationship between risk scores, mortality, and characteristic gene expression (c). Risk score AUC values and clinical indicators at the one-year OS are shown (d).

A prognostic model was next established to evaluate the relevance of *MMP1*, *HMGCS2*, and *SLC27A5* as predictors of HCC patient outcomes based upon the expression of these three genes. Regression coefficients for each gene were obtained through a multivariate Cox regression analysis, yielding the following model: risk score = 0.1488∗expression value of *MMP*1 + (−0.0393)∗expression value of *HMGCS*2 + (−0.0479)∗expression value of *SLC*27*A*5. The predictive efficacy of this model was then assessed by assigning risk scores to 365 HCC patient samples in the TCGA database (Supplemental file 1). In this analysis, patients in the high-risk group exhibited as significantly poorer prognosis relative to patients in the low-risk group (*p* < 0.0001; Figure 3(a)). Specifically, high-risk patients had a median OS of 17.8 months, whereas low-risk patients had a median OS of 22.0 months. Time-dependent ROC analyses were additionally performed to assess 1-, 3-, and 5-year OS, yielding corresponding area under the curve (AUC) values of 0.702, 0.694, and 0.652, consistent with satisfactory model performance (AUC > 0.5; Figure 3(b)). Additionally, high-risk scores were associated with the earlier happened death incident of the patient, coinciding with higher *MMP1* expression and lower *HMGCS2* and *SLC27A5* expression (Figure 3(c)). These data suggested that *MMP1* was not advantageous for the prognosis of HCC, whereas *HMGCS2* and *SLC27A5* were. To compare the prognostic efficacy of our risk scores to other clinical factors, time-dependent ROC analyses were additionally performed based upon patient 1-year OS. Of the analyzed risk factors, risk scores exhibited the best prognostic efficacy, yielding an AUC value of 0.702 (Figure 3(d)). As such, these data indicate that we were able to successfully establish a PPAR-related HCC prognostic model in which *MMP1*, *HMGCS2*, and *SLC27A5* serve as effective predictors of HCC patient outcomes.

### 3.3. Verification of the Prognostic Efficacy of the Multigene Signature

**Figure 4:**
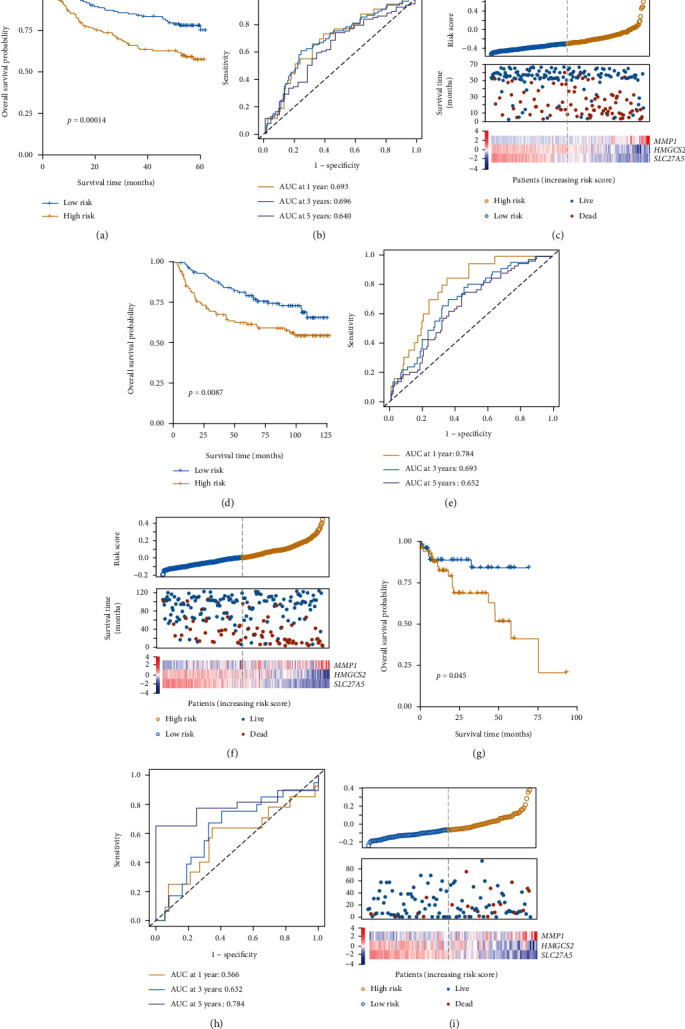
Prognostic model validation. The prognostic efficacy of this model was assessed with the GSE14520 (a–c), GSE36376 (d–f), and GSE76427 (g–i) verification datasets.

To ensure that this multigene signature was not prognostic as a consequence of data overfitting, we validated this signature using three independent datasets GSE14520, GSE36376, and GSE76427. The median OS of patients in the high-risk group (32.8 months in GSE14520 (Figure 4(a)), 63.7 months in GSE36376 (Figure 4(d)), and 11.8 months in GSE76427 (Figure 4(g))) was significantly decreased (*p* = 0.00014, *p* = 0.0087, and *p* = 0.045) relative to that of patients in the low-risk group (53.7 months in GSE14520, 82.7 months in GSE36376, and 16.6 months in GSE76427), consistent with the results from our training dataset. In the three datasets, the AUC values for 1-, 3-, and 5-year OS were 0.693, 0.696, and 0.640 (Figure 4(b)); 0.784, 0.693, and 0.652 (Figure 4(e)); and 0.566, 0.632, and 0.784, respectively (Figure 4(h)). Furthermore, in line with the results from the TCGA cohort, higher risk scores were consistent with the earlier happened patient's death incident and with higher *MMP1* and lower *HMGCS2* and *SLC27A5* expression (Figures 4(c), 4(f), and 4(i)). These results suggest that this PPAR-related risk model was robust across platforms.

### 3.4. The Independence of the Prognostic Multigene Signature

**Figure 5:**
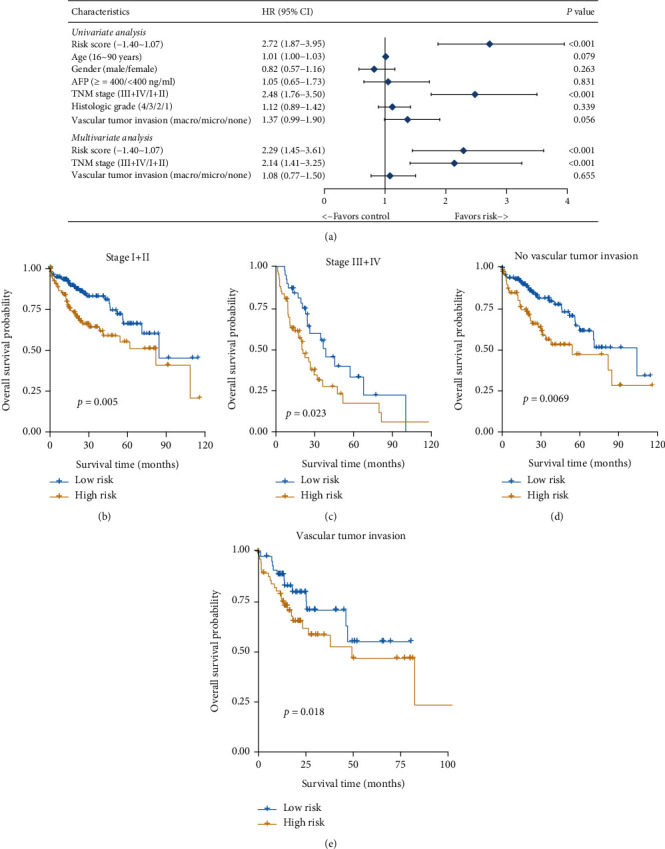
Risk scores are an independent predictor of patient outcomes. Forest plots corresponding to univariate and multivariate Cox regression analyses of the relationship between HCC patient OS and various clinical indicators and risk scores (a). Patients were classified based upon whether they exhibited vascular invasion and based upon their TNM stage. Risk score performance in each patient subcategory was then assessed (b–e).

To confirm the independent predictive value of this multigene signature, we next explored the relationship between HCC patient clinical characteristics, risk score, and outcomes in the TCGA cohort. In univariate Cox regression analyses, TNM stage and risk score were both significantly associated with patient OS (*p* < 0.001). Correlations between vascular tumor invasion and OS approached but did not reach significance (*p* = 0.056). These three factors were then incorporated as covariates in a multivariate Cox regression analysis which revealed both risk score (HR = 2.29, 95%CI = 1.45‐3.61, *p* < 0.001) and TNM stage (HR = 2.14, 95%CI = 1.41‐3.25, *p* < 0.001) to be independent prognostic factors for HCC patient OS (Figure 5(a)). Additionally, we found that whether a patient exhibited early (stage I+II, Figure 5(b)) or advanced (stage III+IV, Figure 5(c)) stage disease and whether or not they exhibited vascular invasion (Figures 5(d) and 5(e)) were predictive of patient survival, underscoring the independent prognostic value of our multigene signature.

### 3.5. Nomogram Establishment and Evaluation

**Figure 6:**
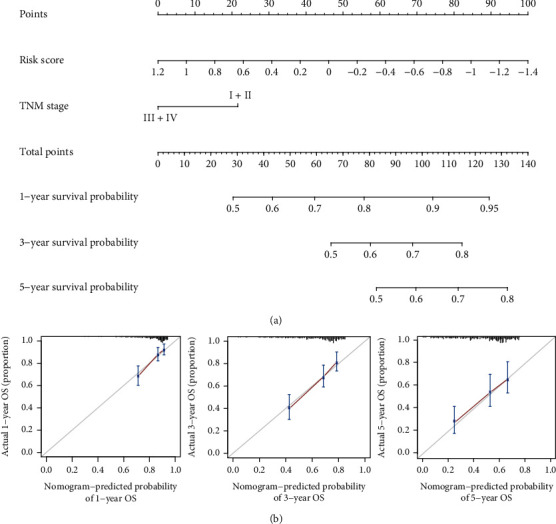
The established nomogram exhibited good predictive ability. This nomogram was generated using a combination of risk scores and TNM stage to predict HCC patient OS (a). Calibration charts corresponding to the prediction of 1-, 3-, and 5-year survival in the training cohort. Horizontal and vertical axes, respectively, correspond to the predicted and actual survival probability (b).

To assess the ability of our model to reliably predict the clinical prognosis of HCC, we next established a nomogram incorporating TNM stage and risk scores as two independent prognostic factors associated with HCC patient 1-, 3-, and 5-year OS (Figure 6(a)). This nomogram yielded a C-index value of 0.709. Calibration plots for all three of these survival time points additionally indicated that the nomogram exhibited good predictive ability (Figure 6(b)). As such, we were able to successfully confirm the reliability and potential clinical value of our multigene signature.

## 4. Discussion

The role of PPARs in the development of cancers including HCC has been revealed by a growing body of research literature [18]. In HCC, current studies on the sensitivity to chemotherapy of PPARs [19] and the correlation between PPARs' target genes and the survival of patients with HCC [20] suggest that finding prognostic markers from PPAR-related genes is more clinically meaningful. Therefore, in this study, to find out the PPAR-related prognostic markers of HCC, we used the HCC patient data collected in TCGA to analyze the 69 genes involved in the PPAR signaling pathway. Finally, the combination of *MMP1*, *HMGCS2*, and *SLC27A5* was screened out as a multigene marker for the prognosis of HCC. The prognostic performance of the marker we selected was good, and the verification in the GEO validation sets shows that there was no sample bias.

In this study, among the three PPAR-related prognostic genes screened, *MMP1* is unfavorable for the prognosis of HCC, while *HMGCS2* and *SLC27A* are favorable (Figure 3(c)). These results were verified by using the Pathology Atlas of the Human Protein Atlas (HPA) database (https://www.proteinatlas.org/). *MMP1* is a member of the matrix metalloproteinase family which has been reported as a risk factor for cancer development [21–23]. In addition, Liao et al. explored the prognostic value of *MMP1* in HCC [24], and the results were consistent with this article. Regarding *HMGCS2*, it is confirmed by researchers as a cancer suppressor [25]. In HCC, the reduction of *HMGCS2* is accompanied by a poor prognosis and promotes cancer cell migration [26]. *SLC27A* is an isozyme of very long-chain acyl-CoA synthetase (VLCS) expressed in the liver. In the current study, the effect of *SLC27A* in HCC or any other cancer has not been reported. In summary, regarding the *MMP1* and *HMGCS2* selected in this article, studies have reported their possible role in the prognosis of HCC. Judging from the current reports, the prognostic genes we selected are relatively reliable. As for *SLC27A*, which has not yet reported its role in HCC or any other cancer, our research presents new possibilities.

## 5. Conclusions

In summary, we found that the PPAR-associated multigene signature selected in this study was able to reliably serve as an independent predictor of HCC prognosis. This signature is robust owing to the cross-platform and cross-batch predictions conducted herein. Overall, our study highlights new potential directions for preclinical research and for the implementation of personalized medicine-based approaches to evaluating HCC patient prognosis and treatment.

## Data Availability

The mRNA and corresponding phenotype data were obtained from UCSC Xena (https://tcga.xenahubs.net/download/TCGA.LIHC.sampleMap/HiSeqV2.gz; https://tcga.xenahubs.net/download/TCGA.LIHC.sampleMap/LIHC_clinicalMatrix) and GEO (GSE14520_GPL3921, GSE36376_GPL10558, and GSE76427_GPL10558).

## Supplementary Materials

Supplementary Materials Supplemental File 1: the risk scores and groupings of 365 HCC samples in TCGA.
