# Supplementary material for: Identification of a Potential PPAR-Related Multigene Signature Predicting Prognosis of Patients with Hepatocellular Carcinoma
Source: PPAR Res. 2021 Mar 12;2021:6642939. doi: 10.1155/2021/6642939 (PMC7981186; doi:10.1155/2021/6642939)
Supplement: Supplementary Materials — Supplemental File 1: the risk scores and groupings of 365 HCC samples in TCGA. [file 6642939.f1.docx]

***Supplementary*** ***Description***

**Supplemental file 1**: The risk scores and groupings of 365 HCC samples in TCGA.
